# Medicinal Plants Used in the Management of Diabetes Mellitus 2015

**DOI:** 10.1155/2015/467196

**Published:** 2015-10-07

**Authors:** Musa T. Yakubu, Taofik O. Sunmonu, Francis B. Lewu, Anofi O. T. Ashafa, Femi J. Olorunniji, Mohamed Eddouks

**Affiliations:** ^1^Phytomedicine, Toxicology and Reproductive Biochemistry Research Laboratory, Department of Biochemistry, University of Ilorin, Ilorin 240003, Nigeria; ^2^Phytomedicine, Environmental Toxicology and Plant Biochemistry Research Laboratory, Department of Biological Sciences, Al-Hikmah University, Ilorin 240001, Nigeria; ^3^Department of Agriculture, Faculty of Applied Sciences, Cape Peninsula University of Technology, Wellington Campus, Wellington, Western Cape 7655, South Africa; ^4^Department of Plant Sciences, University of the Free State, Qwaqwa Campus, Phuthaditjhaba 9866, South Africa; ^5^Institute of Molecular, Cell, and System Biology, College of Medical, Veterinary, and Life Sciences, University of Glasgow, Glasgow G12 8QQ, UK; ^6^Faculty of Sciences and Techniques Errachidia, Moulay Ismail University, 52000 Errachidia, Morocco

Diabetes mellitus is one of the common endocrine disorders prevalent in almost all of the countries. This chronic pathology is characterized by hyperglycemia caused by defective insulin action, insulin secretion, or the combination of both. Prolonged persistence of elevated blood glucose level consequently caused a series of complications such as nephropathy, retinopathy, and cardiomyopathy. Currently available synthetic drugs for treating this disease are found to be associated with many adverse effects. The use of plants in medicine is an age-long practice in various parts of the globe for both preventive and curative purposes. Several warnings have been issued over lack of quality control, scientific evidence for the efficacy, and potential adverse effects of herbal remedies including hepatotoxicity, nephrotoxicity, cardiotoxicity, and reproductive toxicity among others. Despite all of these, reliance on herbs as medicine for the management of diabetes mellitus is still much practiced by a large proportion of the world population because they are readily available and affordable with perceived reduced toxicity. Therefore, with the upsurge of interests in medicinal plants, there is a need for thorough scientific investigations of these plants for both efficacy and potential toxicity. In this issue, we present some recent advances in the use of medicinal plants for treating diabetes mellitus. B. Pang et al. (“Innovative Thoughts of Treating Diabetes from the Perspective of Traditional Chinese Medicine”)presented a review article on the contribution of traditional Chinese medicine to the development of alternative and complementary medicine for the treatment and prevention of diabetes mellitus. In another paper (“Effect of Rhizoma Coptidis (Huang Lian) on Treating Diabetes Mellitus”), B. Pang et al. discussed the efficacy and safety of Rhizoma Coptidis in the treatment of diabetes mellitus. In another study (“Evaluation of the Effects of* Cornus mas* L. Fruit Extract on Glycemic Control and Insulin Level in Type 2 Diabetic Adult Patients: A Randomized, Double-Blind, Placebo-Controlled Clinical Trial”), R. Soltani et al. reported the results of a clinical trial on the effect of* Cornus mas* L. fruit extract on hyperglycemia in type 2 diabetic patients. In addition, W. Liu et al. (“The Effects of Chinese Medicine on Activation of Wnt/*β*-Catenin Signal Pathway under High Glucose Condition”) present a valuable review on some compounds implicated in the regulation of Wnt/*β*-catenin signal pathway as a mechanism of action involved in the antihyperglycemic activity from Chinese medicine. Furthermore, A. O. T. Ashafa and M. I. Kazeem (“Toxicopathological Evaluation of Hydroethanol Extract of* Dianthus basuticus* in Wistar Rats”) reported on the effects of* Dianthus basuticus* (a Basotho plant with acclaimed antidiabetic activity) on some biochemical parameters and histology of Wistar rats. Finally, X.-J. Li et al. (“TCM Formula Xiaoyaosan Decoction Improves Depressive-Like Behaviors in Rats with Type 2 Diabetes”) evaluated the effect of traditional medicine formula, Xiaoyaosan, on the cognitive function of diabetic rats. After the first volume of this special issue that was published in 2014, we hope that this issue will present additional valuable information for scientists and clinicians.

